# Rumination as a mediator between mindfulness and emotion regulation difficulties in female combat sports coaches

**DOI:** 10.3389/fpsyg.2026.1853241

**Published:** 2026-06-17

**Authors:** Mehdi Duyan, Ilker Gunel, Mihriay Musa, Mehmet Bayansalduz, Eyup Ugur

**Affiliations:** 1 Faculty of Sport Sciences, Inonu University, Malatya, Türkiye; 2 School of Physical Education and Sports, Osmaniye Korkut Ata University, Osmaniye, Türkiye; 3 Faculty of Sport Sciences, Istanbul Topkapi University, Istanbul, Türkiye

**Keywords:** difficulties in emotion regulation, mindfulness, rumination, sport psychology, structural equation modeling

## Abstract

**Introduction:**

This study examined the mediating role of rumination in the relationship between mindfulness and difficulties in emotion regulation among female combat-sports coaches.

**Methods:**

A quantitative cross-sectional design was used with 398 female coaches. Data were collected using self-report measures of mindfulness, rumination, and difficulties in emotion regulation. Confirmatory factor analysis and structural equation modeling were conducted using LISREL, and the indirect effect was tested using the Sobel test.

**Results:**

The proposed model demonstrated acceptable fit indices (CFI = 0.95, RMSEA = 0.084, SRMR = 0.076). Mindfulness negatively predicted rumination and difficulties in emotion regulation, whereas rumination positively predicted difficulties in emotion regulation. Rumination partially mediated the relationship between mindfulness and difficulties in emotion regulation. Specifically, higher mindfulness was associated with fewer difficulties in emotion regulation both directly and indirectly through lower rumination.

**Discussion:**

These findings suggest that rumination is an important cognitive mechanism linking mindfulness to difficulties in emotion regulation in high-demand coaching contexts. The study highlights the need to integrate rumination-focused strategies with mindfulness-based approaches in psychological support programs for female combat-sports coaches.

## 1 Introduction

Mindfulness refers to individuals’ capacity to attend to and become aware of their present-moment experiences in a nonjudgmental manner, directing attention deliberately toward these experiences (Baer et al., 2006; Bishop et al., 2004; Kabat-Zinn, 2005). In recent years, mindfulness has been conceptualized as a key self-regulatory resource associated with stress management, psychological well-being, and the maintenance of emotional balance, particularly in contexts characterized by high performance demands (Baer et al., 2006; Kabat-Zinn, 2005). Empirical evidence suggests that mindfulness may enhance psychological resilience and reduce maladaptive cognitive processes among individuals exposed to elevated workload and performance pressure. In contrast, rumination represents a maladaptive cognitive process characterized by repetitive and difficult-to-control focus on negative experiences, emotions, or thoughts (Aldao et al., 2010; Nolen-Hoeksema et al., 2008; Treynor et al., 2003; Watkins, 2008). Ruminative thinking has been consistently linked to sustained stress, emotional exhaustion, and impaired psychological adjustment, particularly in performance-oriented occupations where self-evaluative attention and outcome responsibility are heightened (Treynor et al., 2003). From a cognitive-regulatory perspective, rumination is considered a central mechanism through which emotional distress is maintained and amplified. Emotion regulation refers to individuals’ abilities to recognize, understand, and manage emotional responses in accordance with situational demands (Gratz and Roemer, 2004; Gross, 1998; Gross, 2014). Difficulties in emotion regulation are associated with heightened emotional reactivity, reduced cognitive flexibility, and compromised psychological adjustment under stress (Gratz and Roemer, 2004). Within contemporary emotion regulation frameworks, maladaptive cognitive processes such as rumination are viewed as key contributors to regulatory failure, whereas mindfulness is conceptualized as a higher-order process that can influence emotional responding through attentional and cognitive mechanisms. From this perspective, mindfulness, rumination, and difficulties in emotion regulation can be understood as interrelated psychological processes operating within a shared self-regulatory system. In high-demand performance contexts, however, the protective role of mindfulness may depend on how present-moment awareness is integrated with acceptance and effective cognitive regulation. When mindfulness is accompanied by acceptance and nonjudgmental awareness, it is expected to reduce repetitive negative thinking; however, performance pressure may shape the strength and expression of this protective association. Therefore, examining rumination as a mediating mechanism may help clarify how mindfulness is linked to difficulties in emotion regulation in demanding coaching contexts (Dahl et al., 2015; Graen and Uhl-Bien, 1995). This interplay highlights the importance of examining rumination as a potential mechanism through which mindfulness is associated with difficulties in emotion regulation. Female combat sports coaches operate in professional environments characterized by intense physical and psychological demands. Combat sports inherently involve continuous performance pressure, competitive evaluation, and demanding training schedules. In addition to these sport-specific stressors, female coaches often encounter gender-based biases, role conflict, and limited professional visibility, which may further intensify cognitive and emotional strain (Burton, 2015; LaVoi and Dutove, 2012; Norman, 2014). These multidimensional stressors may directly influence self-regulatory processes by increasing ruminative thinking, reducing effective mindfulness, and exacerbating difficulties in emotion regulation. Although the existing literature generally reports an inverse relationship between mindfulness and rumination (Baer et al., 2006; Chambers et al., 2008; Hill and Updegraff, 2012), associations between mindfulness and fewer difficulties in emotion regulation (Baer et al., 2006; Hill and Updegraff, 2012), most prior research has relied on bivariate approaches. Moreover, studies within sport psychology examining these constructs have predominantly focused on male or mixed-gender samples, with limited attention given to female coaches, particularly in high-stress contexts such as combat sports (Josefsson et al., 2017; Wagstaff et al., 2025; Moulds and McEvoy, 2025; Bicalho and Costa, 2018; Nicholls et al., 2009). Comprehensive models simultaneously examining mindfulness, rumination, and difficulties in emotion regulation using structural modeling approaches remain scarce (Aldao et al., 2010; Hayes, 2017). Given these gaps, the present study aimed to examine the mediating role of rumination in the relationship between mindfulness and difficulties in emotion regulation among female combat sports coaches, using a structural equation modeling approach. Furthermore, research simultaneously examining mindfulness, rumination, and difficulties in emotion regulation among female combat sports coaches using structural equation modeling remains relatively limited, and these relationships may extend beyond sport contexts as core psychological processes relevant to broader high-demand occupational settings. Direct and indirect associations among the study variables were tested using structural equation modeling (Aldao et al., 2010; Hayes, 2017). The following hypotheses were tested in the present study:

*H_1_*: Mindfulness significantly predicts levels of rumination among female combat sports coaches.

*H_2_*: Rumination significantly predicts difficulties in emotion regulation among female combat sports coaches.

*H_3_*: Mindfulness significantly predicts difficulties in emotion regulation among female combat sports coaches.

*H_4_*: Rumination mediates the relationship between mindfulness and difficulties in emotion regulation among female combat sports coaches.

## 2 Method

### 2.1 Population and sample

This study was conducted using a quantitative research design based on the correlational survey method, which is commonly employed to examine the relationships between two or more variables (Gürbüz and Şahin, 2014). The study sample consisted of 398 female combat-sports coaches working in the Provincial Directorates of Youth and Sports and the Taekwondo, Muay Thai, Kickboxing, and Boxing Federations in the provinces of Istanbul, Antalya, and Izmir. The sampling frame was created by identifying eligible female coaches registered with or actively working through the Provincial Directorates of Youth and Sports and the relevant Taekwondo, Muay Thai, Kickboxing, and Boxing Federations in Istanbul, Antalya, and Izmir. These provinces and sport branches were selected because they provided access to a sufficiently large number of active female combat-sports coaches within the scope of the study. After the eligibility criteria were applied, coaches in the accessible sampling frame were assigned identification numbers. Potential participants were then randomly selected from these lists and invited to participate in the study. Coaches who agreed to participate, met the inclusion criteria, and provided informed consent were included in the final sample. This procedure was used to reduce selection bias within the accessible population of female combat-sports coaches. Inclusion criteria included actively working as a coach, having at least 1 year of coaching experience, and providing informed consent to participate in the study. Participants with incomplete questionnaire responses were excluded from the analyses. Demographic characteristics of the participants, including age, marital status, educational level, coaching experience, sports branch, and weekly training frequency, are presented in Table 1. The decision to include only female combat sports coaches in the present study was based on both theoretical and contextual considerations. Combat sports have historically been characterized as male-dominated environments, in which norms related to physical strength, aggression, and competitiveness are often constructed through masculinized cultural expectations. Within this context, female coaches may be exposed to a distinct set of psychosocial stressors that differ from those experienced by their male counterparts (Norman, 2014; de Haan and Knoppers, 2020). Previous research has shown that women working as coaches frequently report greater role conflict, heightened pressure to demonstrate professional competence, limited institutional support, and increased emotional labor compared to male coaches (Burton, 2015; LaVoi and Dutove, 2012). These challenges may be further intensified in combat sports, where men are numerically and culturally dominant, potentially amplifying gender-based scrutiny and workload demands placed on female coaches. Given these contextual factors, psychological processes such as mindfulness, rumination, and difficulties in emotion regulation may operate differently for female coaches than for male coaches. Including both genders within the same analytical model could obscure these gender-specific dynamics. Therefore, a homogeneous sample of female combat sports coaches was deliberately selected to allow for a more focused examination of the relationships among the study variables. This approach is consistent with prior research emphasizing the importance of gender-sensitive analyses in sport and coaching contexts and contributes to the limited body of literature specifically addressing the psychological experiences of women coaches.

**Table 1:** Demographic characteristics of the participants.

Variables	Categories	f	%	x̄/sd
Age	24–27	214	53.8	28.20 ± 3.47
	28–31	122	30.7	
	32–35	36	9.0	
	36 years and above	26	6.5	
Marital status	Married	273	68.6	
	Single	125	31.4	
Coaches’ branch	Boxing	59	14.8	
	Taekwondo	102	25.6	
	Muay Thai	135	33.9	
	Kickboxing	102	25.6	
Educational status	Undergraduate	287	72.1	
	Master’s/Doctorate	111	27.9	
Coaching experience	1–2 year	93	23.4	Coaching Experience (years $\bar{x}$ /sd) 3.88 ± 1.60
	3–4 year	122	30.7	
	5–6 year	167	42.0	
	9 year and over	16	4.0	
Prior athletic background	Yes	304	76.4	
	No	94	23.6	
Weekly training frequency	At least 3 days	241	60.6	
	4–5 days	102	25.6	
	6–7 days	55	13.8	

*N* = 398; *N*. Total number of participants.

### 2.2 Data collection instruments

#### 2.2.1 Mindfulness scale

Mindfulness was assessed using the Mindfulness Scale, which measures individuals’ awareness and attention to present-moment experiences. The scale was originally developed by Brown and Ryan (Brown and Ryan, 2003) and subsequently adapted into Turkish by Ozyesil, Arslan (Ozyesil et al., 2011). It consists of 12 items rated on a Likert-type scale. In the present study, the internal consistency of the scale was found to be high (Cronbach’s *α* = 0.92).

#### 2.2.2 Rumination scale

Rumination was assessed using a scale developed by Treynor, Gonzalez (Treynor et al., 2003) to evaluate repetitive and intrusive thought patterns, which was adapted into Turkish by Erdur-Baker and Bugay (Erdur-Baker and Bugay, 2010). The scale consists of 10 items rated on a Likert-type response format, with higher scores indicating greater levels of rumination. In the present study, the scale demonstrated high internal consistency, with a Cronbach’s alpha coefficient of 0.86.

#### 2.2.3 Difficulties in emotion regulation scale (DERS)

Difficulties in emotion regulation were assessed using the Difficulties in Emotion Regulation Scale (DERS) developed by Gratz and Roemer (Gratz and Roemer, 2004). The Turkish adaptation of the scale was conducted by Rugancı and Gençöz (Rugancı and Gençöz, 2010). Items are rated on a Likert-type scale, with higher scores indicating greater difficulties in emotion regulation. In the present study, the scale demonstrated satisfactory internal consistency, with a Cronbach’s alpha coefficient of 0.79.

### 2.3 Data collection procedure

Data were collected through an electronic survey administered to participants during scheduled training sessions. Prior to data collection, participants were provided with a written explanation outlining the purpose of the study. Due to the scientific nature of the research, confidentiality and anonymity were assured. Informed consent was obtained from all participants before participation. Participation in the study was entirely voluntary, and participants were informed of their right to withdraw from the study at any time without any consequence.

### 2.4 Ethical approval

This study was approved by the Health Sciences Research Ethics Committee of Osmaniye Korkut Ata University (Decision No: 2026/3/10, Date: 02 March 2026). All procedures performed in this study involving human participants were conducted in accordance with the ethical standards of the institutional research committee and with the 1964 Helsinki Declaration and its later amendments. Informed consent was obtained from all participants prior to their participation in the study.

### 2.5 Statistical analysis

Data analyses were conducted using IBM SPSS Statistics 26.0 for descriptive statistics, reliability analyses, normality testing, correlation analyses, and *t*-tests. Normality was assessed using skewness and kurtosis values, with coefficients within the ±1.96 range considered indicative of a normal distribution. Confirmatory factor analysis (CFA) and structural equation modeling (SEM) were performed using LISREL 8.71. Model fit was evaluated using multiple fit indices, including the chi-square to degrees of freedom ratio (χ^2^/df), root mean square error of approximation (RMSEA), comparative fit index (CFI), normed fit index (NFI), non-normed fit index (NNFI), and standardized root mean square residual (SRMR). To examine the mediating role of rumination in the relationship between mindfulness and difficulties in emotion regulation, a mediation model was tested using SEM. To improve model parsimony and reduce model complexity, item parceling was applied by combining items into theoretically meaningful indicators. Parceling is commonly used in structural equation modeling to enhance model stability, reduce random measurement error, and achieve more reliable parameter estimates, particularly in models with a relatively large number of observed variables (Little et al., 2002; Matsunaga, 2008). Direct, indirect, and total effects were estimated based on standardized path coefficients. Direct, indirect, and total effects were estimated based on standardized path coefficients. The significance of the indirect effect was assessed using the Sobel test rather than bootstrap resampling. This decision was made because the mediation model was estimated within the LISREL framework, and the Sobel test allows the indirect effect to be evaluated using the unstandardized path coefficients and their standard errors. We acknowledge that bootstrap confidence intervals are generally recommended in contemporary mediation analysis because the sampling distribution of indirect effects may deviate from normality. However, the Sobel test remains a commonly used parametric approach when the sample size is sufficiently large, the measurement model demonstrates acceptable fit, and the component paths of the indirect effect are estimated with adequate precision (Hayes, 2017; Kline, 2023; MacKinnon et al., 2002). In the present study, the sample size was relatively large (*N* = 398), the relevant path coefficients were statistically significant, and the structural model demonstrated acceptable fit, supporting the use of the Sobel test as a reasonable inferential procedure. Nevertheless, because bootstrapped confidence intervals may provide more robust evidence for indirect effects, the absence of bootstrapped confidence intervals was considered when interpreting the mediation findings. In the present study, the mediation model was evaluated within a traditional covariance-based SEM framework, and the Sobel test was preferred as a theory-driven and parsimonious inferential approach consistent with this analytical strategy. Future studies may benefit from complementing these findings with bootstrap-based estimation procedures to provide additional robustness for indirect effect testing. Statistical significance was set at *p* < 0.05. Age and coaching experience were included in the analyses as continuous proxy variables based on the midpoint of each category. In addition, a *post hoc* power analysis was conducted using G*Power 3.1. For a linear multiple regression model (fixed model, *R*^2^ deviation from zero), assuming a medium effect size (*f*^2^ = 0.15), an alpha level of 0.05, two predictors, and a total sample size of 398, the achieved statistical power was 1.00. This indicates that the sample size was more than sufficient to detect the hypothesized effects (see Table 1).

The study sample consisted of 398 female combat sports coaches. More than half of the participants were between the ages of 24 and 27 years (53.8%), followed by those aged 28–31 years (30.7%), the mean age of the participants was 28.20 ± 3.47 years. The majority of the participants were married (68.6%). Regarding sport discipline, participants were distributed across boxing (14.8%), taekwondo (25.6%), Muay Thai (33.9%), and kickboxing (25.6%). Most of the coaches held a bachelor’s degree (72.1%), while 27.9% had completed postgraduate education. In terms of coaching experience, 42.0% of the participants reported 5–6 years of experience, followed by 3–4 years (30.7%) and 1–2 years (23.4%). A substantial proportion of the sample reported having a prior athletic background (76.4%). Additionally, 60.6% of the participants reported training at least 3 days per week (see Table 2).

**Table 2:** Descriptive statistics and normality indicators of the study variables.

Scales	x̄	SD	Median	Variance	Skewness	Kurtosis
Mindfulness	53.30	6.35	54.00	40.44	−1.179	2.577
DERS	33.50	5.50	34.00	25.31	−1.012	1.631
Rumination	35.59	6.10	38.50	36.14	−0.280	−0.080

The close proximity of the mean and median values, acceptable skewness and kurtosis coefficients (George and Mallery, 1999), and visual inspection of Q–Q plots collectively indicated that the data exhibited acceptable univariate normality. Accordingly, the use of parametric tests was considered appropriate for the analyses (see Table 3).

**Table 3:** Independent samples *t*-test results according to prior athletic background.

Scales	Prior athletic background	Levene test							
		*n*	x̄	sd	*F*	*p*	*t*	df	*p*
M	Yes	304	53.5	6.39	0.495	0.482	1.139	396	0.256
	No	94	52.65	6.25					
DERS	Yes	304	33.5	5.03	0.038	0.845	−0.041		0.967
	No	94	33.52	5.05					
R	Yes	304	38.92	6.1	2.135	0.145	1.985		**0.040** ^*^
	No	94	37.52	5.61					

^*^p < 0.05. M, mindfulness; R, rumination; DERS, difficulties in emotion regulation.

Independent samples *t*-tests indicated no significant differences between coaches with and without a prior athletic background in terms of mindfulness, *t*_(396)_ = 1.14, *p* = 0.256, or difficulties in emotion regulation, *t*_(396)_ = −0.04, *p* = 0.967. However, a significant difference was observed for rumination, with coaches who had a prior athletic background reporting higher rumination levels than those without such a background, *t*_(396)_ = 1.99, *p* = 0.040, although the effect size was small (Cohen’s d = 0.23) (see Table 4).

**Table 4:** Internal consistency reliability of the study scales.

Scale	Number of items	Cronbach’s
Mindfulness	12	0.92
Rumination	10	0.86
Difficulties in emotion regulation	8	0.79

The internal consistency of the study scales ranged from acceptable to excellent. Cronbach’s alpha coefficients were 0.92 for mindfulness, 0.86 for rumination, and 0.79 for difficulties in emotion regulation, indicating that all measures demonstrated sufficient reliability for subsequent analyses (see Table 5).

**Table 5:** Pearson correlation coefficients among study variables.

Variable	1	2	3	4	5
1. Age	1				
2. Coaching experience	0.61^**^	1			
3. Mindfulness	0.02	0.11^*^	1		
4. Difficulties in emotion regulation	−0.00	−0.05	−0.44^**^	1	
5. Rumination	−0.02	−0.07	−0.25^**^	0.40^**^	1

^*^p < 0.05. ^**^p < 0.01.

Pearson correlation analyses were conducted to examine the relationships among age, coaching experience, mindfulness, rumination, and difficulties in emotion regulation. The results indicated that mindfulness was significantly and negatively correlated with rumination (*r* = −0.25, *p* < 0.01) and difficulties in emotion regulation (*r* = −0.44, *p* < 0.01). In addition, rumination showed a moderate and positive association with difficulties in emotion regulation (*r* = 0.40, *p* < 0.01). Coaching experience was weakly but significantly correlated with mindfulness (*r* = 0.11, *p* < 0.05), whereas age was not significantly associated with mindfulness, rumination, or difficulties in emotion regulation (*p* > 0.05). Overall, the observed correlation pattern was consistent with the theoretical assumptions underlying the proposed mediation model (see Table 6).

**Table 6:** Standardized factor loadings and t-values for the three-factor CFA model.

Scales	Items	Standardized loadings	*t*-value	AVE	CR
Mindfulness	M1	0.66	14.52	0.50	0.92
	M2	0.67	14.68		
	M3	0.77	17.74		
	M4	0.74	16.96		
	M5	0.75	17.19		
	M6	0.74	16.80		
	M7	0.69	15.29		
	M8	0.72	16.17		
	M9	0.72	16.05		
	M10	0.73	16.46		
	M11	0.72	16.06		
	M12	0.60	12.65		
Difficulties in emotion regulation	D1	0.47	9.26	0.32	0.80
	D2	0.66	14.02		
	D3	0.71	15.46		
	D4	0.77	17.03		
	D5	0.78	17.69		
	D6	0.66	14.11		
	D7	0.67	14.30		
	D8	0.72	15.74		
Rumination	R1	0.29	5.41	0.47	0.87
	R2	0.31	5.95		
	R3	0.60	12.38		
	R4	0.33	6.41		
	R5	0.77	17.09		
	R6	0.81	18.54		
	R7	0.27	5.19		
	R8	0.70	14.99		
	R9	0.70	15.00		
	R10	0.45	8.88		

As shown in Table 6, standardized factor loadings obtained from the confirmatory factor analysis ranged from 0.60 to 0.77 for mindfulness, 0.47 to 0.78 for emotion regulation difficulties, and 0.27 to 0.81 for rumination. All factor loadings were statistically significant, with t-values exceeding the critical value of 1.96, indicating that all observed indicators contributed significantly to their respective latent constructs. However, several rumination indicators showed relatively low standardized loadings, particularly R1, R2, and R7. These items were retained for theoretical and measurement-related reasons. First, they represent conceptually relevant aspects of repetitive negative thinking and are part of the validated Turkish version of the rumination scale. Second, removing individual items solely on the basis of lower factor loadings could reduce the content coverage of the construct and compromise comparability with previous studies using the same measure. Third, despite the presence of low-loading items, the rumination construct demonstrated satisfactory internal consistency and composite reliability, indicating that the latent construct was adequately represented at the scale level.

Composite reliability (CR) and average variance extracted (AVE) values were calculated to assess convergent validity. All constructs demonstrated satisfactory composite reliability values exceeding the recommended threshold of 0.70. The AVE value for rumination was slightly below the ideal threshold of 0.50 but remained close to this criterion, suggesting that although some indicators contributed less strongly to the latent construct, the overall reliability of the rumination factor was acceptable. Therefore, the low-loading rumination items were retained to preserve the theoretical integrity and content validity of the measure. The overall three-factor CFA model also demonstrated acceptable fit, suggesting that retaining these items did not substantially compromise the adequacy of the measurement model. Although several rumination indicators demonstrated relatively low standardized factor loadings, the decision to retain these items was theoretically driven. Exploratory consideration was given to the potential removal of low-loading items; however, because these indicators represent conceptually relevant aspects of repetitive negative thinking and belong to the validated Turkish adaptation of the scale, item removal was not prioritized. Moreover, the overall CFA model demonstrated acceptable fit and the rumination construct showed satisfactory internal consistency and composite reliability. Future studies may further compare full and reduced measurement models to examine whether excluding lower-loading indicators improves measurement precision without compromising content validity.

**Figure 1:**
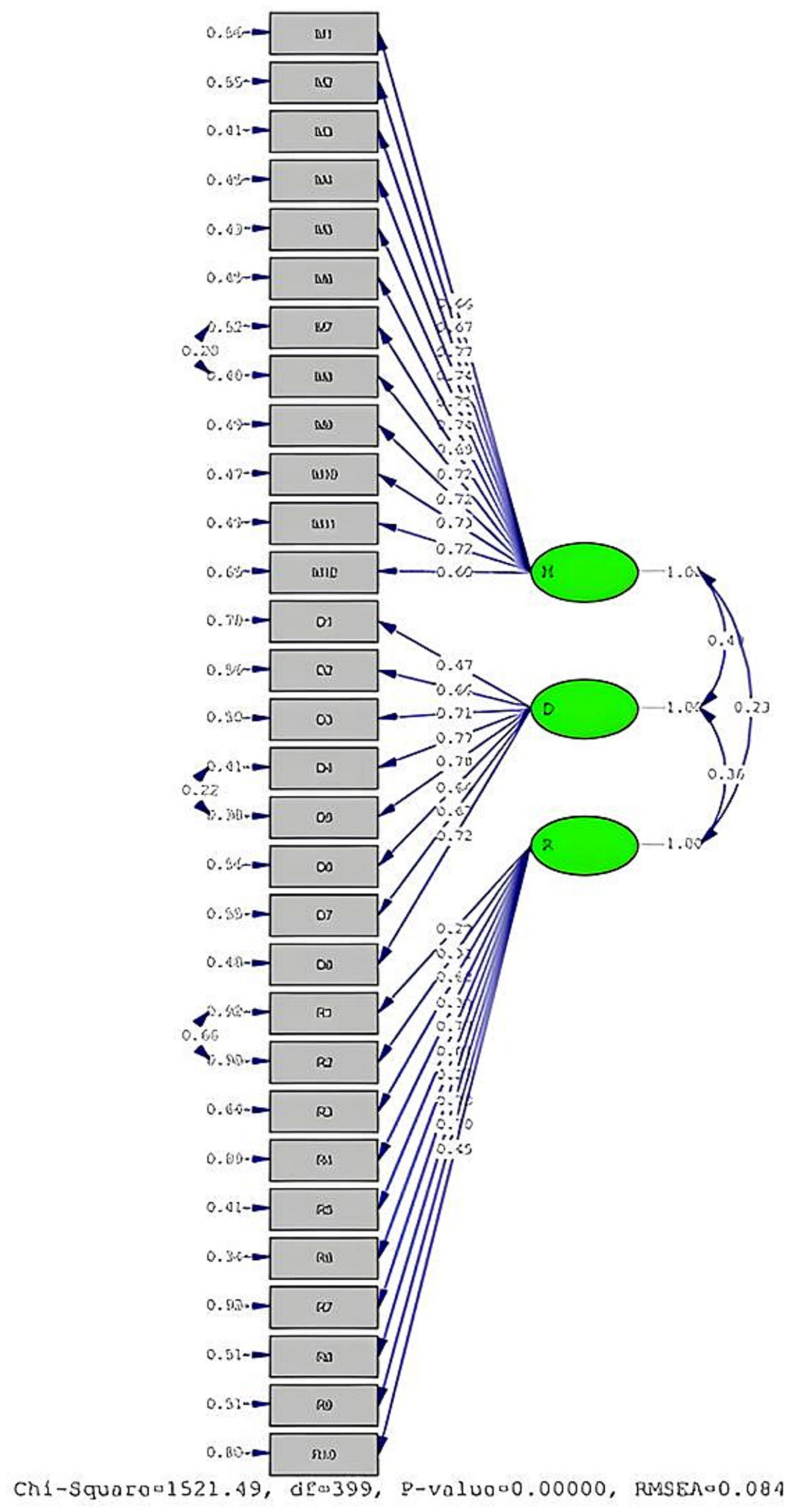
Confirmatory factor analysis (CFA) model of the three-factor measurement model. (D, difficulties in emotion regulation; R, rumination; M, mindfulness).

The AVE value for difficulties in emotion regulation was also below the conventional 0.50 threshold, indicating that convergent validity for this construct may be weaker than ideal. However, this construct was retained because its composite reliability exceeded the recommended 0.70 threshold and all factor loadings were statistically significant. This suggests that although the indicators collectively demonstrated acceptable reliability, the amount of variance captured by the latent construct relative to measurement error was limited. Therefore, findings involving difficulties in emotion regulation should be interpreted with this measurement consideration in mind (see Figure 1).

A confirmatory factor analysis (CFA) was conducted to evaluate the three-factor measurement model comprising mindfulness, rumination, and difficulties in emotion regulation. The results indicated that the measurement model demonstrated an acceptable fit to the data (χ^2^ = 1521.49; df = 399; χ^2^/df = 3.81). Incremental fit indices suggested good model fit (CFI = 0.95; NFI = 0.92; NNFI/TLI = 0.94). While the absolute fit indices were within acceptable limits (RMSEA = 0.084; SRMR = 0.076). Overall the CFA findings supported the adequacy of the proposed three-factor measurement model for subsequent structural equation modeling analyses. Structural equation modeling revealed that mindfulness significantly predicted rumination (*a* = −0.39, SE = 0.051, *t* = 7.58, *p* < 0.001), and rumination significantly predicted difficulties in emotion regulation (*b* = 0.32, SE = 0.053, *t* = 6.00, *p* < 0.001). Mindfulness also had a significant direct effect on difficulties in emotion regulation (c′ = −0.38, SE = 0.058, *t* = 6.57, *p* < 0.001). The indirect effect of mindfulness on difficulties in emotion regulation through rumination was significant and negative (indirect effect = −0.125; Sobel *Z* = −4.74; *p* < 0.001), indicating that higher mindfulness was associated with fewer difficulties in emotion regulation partly through lower levels of rumination.

### 2.6 SEM/mediation results

**Figure 2:**
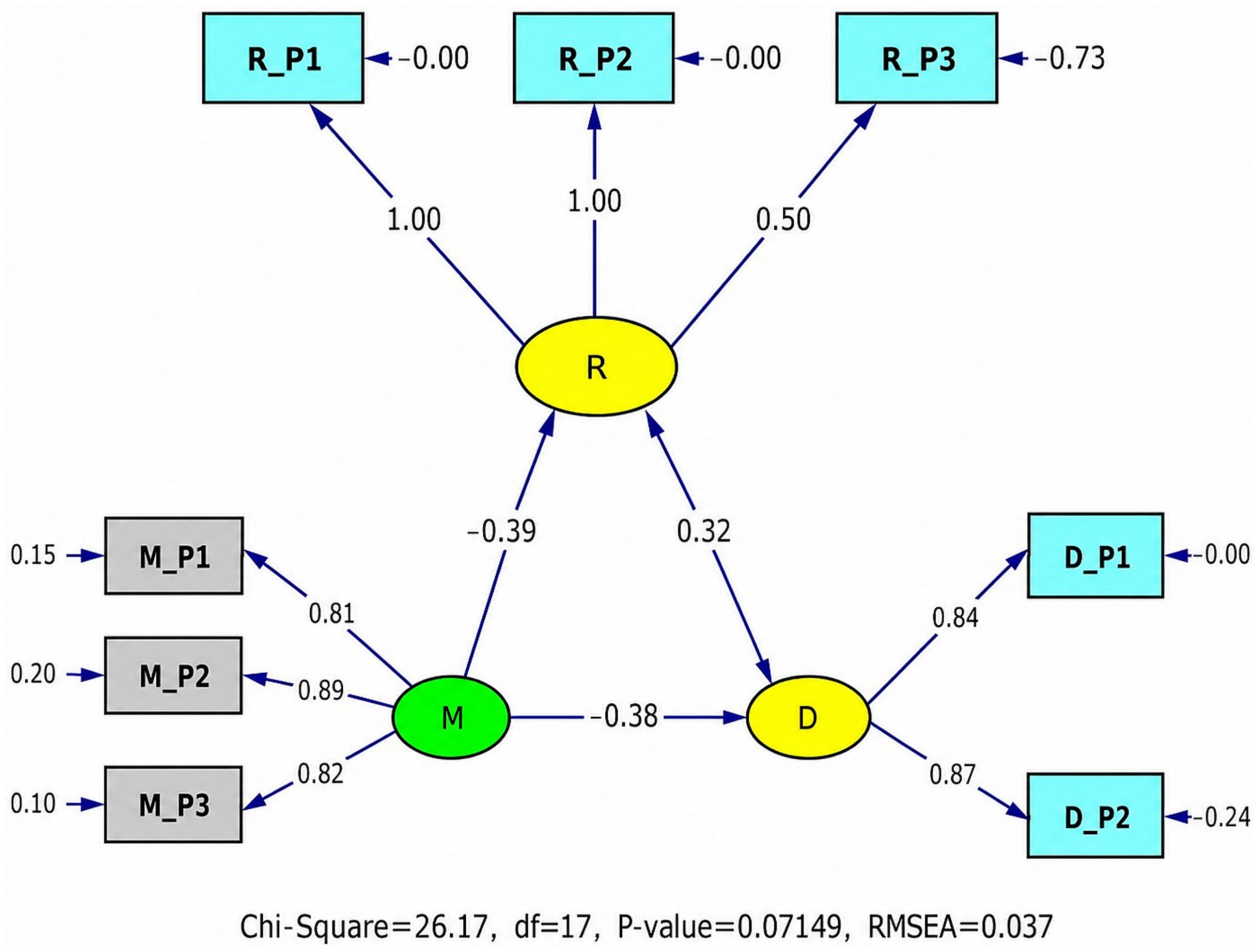
Structural equation model illustrating the mediating role of rumination in the relationship between mindfulness and difficulties in emotion regulation. Standardized path coefficients are presented.

Structural equation model illustrating the mediating role of rumination in the relationship between mindfulness and difficulties in emotion regulation. Standardized path coefficients are presented. All displayed paths are statistically significant (*p* < 0.001) (see Figure 2; Table 7).

**Table 7:** Direct, indirect, and total effects in the mediation model examining the relationship between mindfulness, rumination, and difficulties in emotion regulation.

Path	*β*	*t*-value
Mindfulness → rumination	−0.39	7.58
Rumination → DERS	0.32	6.00
Mindfulness → DERS (direct)	−0.38	6.57
Indirect effect (M → R → D)	−0.13	–
Total effect	−0.51	–

Unstandardized coefficients are reported in the text and Sobel test, whereas standardized coefficients (*β*) are presented in Table 7.

**Figure 3:**
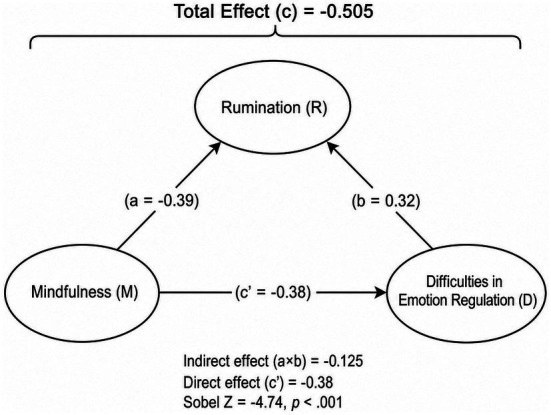
Structural equation model illustrating the mediating role of rumination in the relationship between mindfulness and difficulties in emotion regulation. Unstandardized path coefficients are presented. The significance of the indirect effect was tested using the Sobel test.

*β* values represent standardized path coefficients obtained from the structural equation model. Mindfulness significantly and negatively predicted rumination (*β* = −0.39; *t* = 7.58; *p* < 0.001). explaining 15% of the variance in rumination. Rumination had a significant positive effect on difficulties in emotion regulation (*β* = 0.32; *t* = 6.00; *p* < 0.001). While Mindfulness also had a significant negative direct effect on difficulties in emotion regulation (*β* = −0.38; *t* = 6.57; *p* < 0.001). The indirect effect of mindfulness on difficulties in emotion regulation through rumination was −0.13, yielding a total effect of −0.51. This finding indicates that mindfulness was associated with fewer difficulties in emotion regulation both directly and indirectly through reduced rumination, supporting a partial mediation model. The significance of the indirect effect was confirmed using the Sobel test (*Z* = −4.74; *p* < 0.001). All statistical analyses were conducted using SPSS and LISREL (see Figure 3).

## 3 Discussion

Building on the structural equation modeling results, the present discussion interprets the observed direct and indirect pathways within the broader theoretical and applied sport psychology literature. The present study provides evidence that rumination partially mediates the relationship between mindfulness and difficulties in emotion regulation among female combat-sports coaches. Specifically, higher mindfulness was associated with lower rumination and fewer difficulties in emotion regulation, whereas higher rumination was associated with greater difficulties in emotion regulation. This pattern is consistent with theoretical accounts positioning mindfulness as a self-regulatory resource and rumination as a cognitive vulnerability linked to dysregulated affective responding (Aliche and Idemudia, 2025; Atta et al., 2024; Nolen-Hoeksema, 2012; Feurer et al., 2021; Perestelo-Perez et al., 2017). Emerging neuroscientific evidence further suggests that mindfulness training may alter brain dynamics underlying ruminative processes, indicating that the mindfulness–rumination link reflects both psychological and neurobiological mechanisms (van der Velden et al., 2023). The mediating role of rumination should be interpreted within the performance-driven context of combat-sports coaching, where continuous self-monitoring, evaluative attention, and responsibility for competitive outcomes are central features of professional functioning (Birrer et al., 2012; Henriksen et al., 2019; Olusoga et al., 2019; Sarkar and Fletcher, 2013). In such contexts, the protective association between mindfulness and rumination may be shaped by contextual pressures and may depend on whether awareness is accompanied by acceptance, self-compassion, and effective cognitive regulation strategies (van der Velden et al., 2023; Britton, 2019; Bühlmayer et al., 2017; Hut et al., 2021; Lindsay and Creswell, 2017). Recent empirical and theoretical work also suggests that, in achievement-oriented environments, rumination may arise from sustained attentional focus and self-evaluative processing rather than reflecting a purely general maladaptive tendency (Nolen-Hoeksema et al., 2008; Whitmer and Gotlib, 2013; Koster et al., 2011). In addition, evidence from sport and occupational psychology indicates that female coaches may experience heightened emotional labor, performance scrutiny, and gendered expectations, all of which can amplify cognitive load and emotional strain (Olusoga et al., 2019; Allen and Shaw, 2013; Bentzen et al., 2016; Hägglund et al., 2024). Accordingly, rumination may represent a context-sensitive cognitive pathway through which professional demands are linked to difficulties in emotion regulation among female combat-sports coaches. These findings also clarify that mindfulness did not increase rumination in the present model; rather, mindfulness functioned primarily as a protective factor, with higher mindfulness being associated with lower rumination and fewer difficulties in emotion regulation (Nolen-Hoeksema et al., 2008; van der Velden et al., 2023; Birrer et al., 2012; Henriksen et al., 2019; Olusoga et al., 2019; Sarkar and Fletcher, 2013; Britton, 2019; Bühlmayer et al., 2017; Hut et al., 2021; Lindsay and Creswell, 2017; Whitmer and Gotlib, 2013).

From an applied perspective, these findings suggest that mindfulness-based approaches, when implemented in isolation, may not consistently produce the expected regulatory benefits among groups exposed to high cognitive and emotional demands, such as female combat-sports coaches (Kazdin, 2017; Kuyken et al., 2015). Furthermore, the magnitude of the standardized structural coefficients suggested that mindfulness demonstrated a moderate direct association with emotion regulation difficulties, whereas the indirect pathway through rumination reflected a smaller but meaningful mediating effect. These findings support the practical relevance of repetitive negative thinking as an explanatory cognitive mechanism in high-performance coaching contexts. Applied literature highlights that mindfulness interventions may be more effective when integrated with broader psychological support frameworks, including coping skills training and organizational support to mitigate job stress, strain, and burnout among coaches (Kang and Lee, 2025). Research focused on women sports coaches further shows that job-related stressors, such as workload, performance expectations, and professional strain, are closely linked with psychological well-being outcomes, suggesting that interventions should address these demands holistically rather than targeting mindfulness alone (Didymus et al., 2021; Simpson et al., 2024). In practice, psychological skills training that includes cognitive restructuring, emotional labor strategies, and social support mechanisms may help reduce cognitive load during performance pressure, transform self-critical inner dialogue following mistakes, and redirect repetitive negative thinking toward functional problem-solving (Ha et al., 2021). A broader systematic review also indicates that elite coaches frequently experience psychological difficulties arising from performance pressure, excessive workloads, and social expectations, all of which can diminish coaching effectiveness if not properly supported (Kang and Lee, 2025; Gu et al., 2015). Therefore, positioning rumination as a central target within mindfulness-based coaching programs—and embedding these programs within comprehensive stress-management and organizational support structures—may play a critical role in reducing difficulties in emotion regulation and improving coaches’ overall psychological well-being.

## 4 Conclusion and practical implications

This study demonstrates that rumination partially mediates the relationship between mindfulness and difficulties in emotion regulation among female combat-sports coaches. The findings indicate that mindfulness functions primarily as a protective factor, as higher mindfulness was associated with lower rumination and fewer difficulties in emotion regulation. At the same time, this protective association appears to be context-sensitive, suggesting that mindfulness-based approaches may be most effective when combined with strategies targeting rumination, acceptance, self-compassion, and cognitive flexibility.

From a practical perspective, coach education programs should include specific modules aimed at helping coaches recognize, monitor, and manage ruminative thinking. Such modules may include brief psychoeducation on the difference between constructive post-performance reflection and repetitive self-critical rumination, structured reflective exercises after training sessions or competitions, mindfulness practices combined with acceptance and self-compassion techniques, and cognitive reappraisal strategies that help coaches redirect attention from uncontrollable outcomes toward controllable coaching behaviors. In addition, coach education programs may benefit from incorporating group-based discussions, peer support activities, and referral pathways to psychological support services, particularly for female coaches working in highly evaluative and male-dominated combat-sport environments. Overall, the study advances a more nuanced and context-sensitive understanding of mindfulness in sport psychology by positioning rumination as a central cognitive mechanism linking mindfulness to difficulties in emotion regulation. Interventions that integrate mindfulness with rumination-focused cognitive strategies may help coaches better manage performance-related stress and emotional demands. For example, coach education programs could incorporate brief mindfulness exercises combined with guided reflective practices aimed at distinguishing constructive performance evaluation from repetitive self-critical rumination. In addition, structured group discussions, self-compassion training, and cognitive reappraisal exercises may help coaches develop healthier responses to competitive pressure, performance setbacks, and emotionally demanding coaching situations.

### 4.1 Strengths and limitations

A key strength of the present study lies in its use of structural equation modeling to examine the cognitive mechanisms underlying the relationship between mindfulness and difficulties in emotion regulation. This approach allowed the simultaneous estimation of direct and indirect effects while accounting for measurement error. The focus on female combat-sports coaches represents an additional strength, as this group remains underrepresented in sport psychology research despite being exposed to high cognitive, emotional, and performance-related demands. Moreover, the integration of rumination as a mediating variable provides a more nuanced and mechanism-based understanding of mindfulness in high-performance coaching contexts. Another methodological strength is that item parceling was not applied to artificially improve model fit, but rather to achieve a more parsimonious and stable measurement model consistent with recommendations in the SEM literature.

Several limitations should also be acknowledged. First, the cross-sectional design precludes causal inferences regarding the temporal ordering of mindfulness, rumination, and difficulties in emotion regulation. Therefore, the findings should be interpreted with caution, as the study design does not allow firm conclusions regarding the directionality of the observed relationships. Second, reliance on self-report measures may have introduced response biases, including social desirability and common method variance. Third, several rumination indicators showed relatively low standardized factor loadings. Although these items were retained to preserve the theoretical breadth and validated structure of the measure, their presence may have contributed to the lower AVE value for rumination. Future studies should further examine the dimensional structure of the rumination measure in coaching populations and compare full and reduced measurement models. Fourth, the AVE value for difficulties in emotion regulation was below the conventional 0.50 threshold. Although this construct demonstrated acceptable composite reliability, the lower AVE suggests that convergent validity may be weaker than ideal. Therefore, findings involving difficulties in emotion regulation should be interpreted with appropriate caution, and future studies should further examine the measurement properties of this construct in coaching populations. In addition, the relatively low AVE values may indicate that the rumination and emotion regulation constructs capture heterogeneous cognitive and emotional experiences that are influenced by multiple contextual and interpersonal factors in high-performance coaching environments. Although convergent validity estimates were lower than ideal, acceptable composite reliability coefficients and satisfactory overall model fit supported the retention and theoretical interpretation of these constructs within the structural model. Finally, the sample was limited to female coaches within combat sports, which may restrict the generalizability of the findings to other coaching populations, male coaches, or different sport contexts. Future research employing longitudinal or intervention-based designs across diverse coaching samples would be valuable in extending the present findings.

## Data Availability

The original contributions presented in the study are included in the article/supplementary material, further inquiries can be directed to the corresponding author.
